# The Cancer Stem Cell Concept in Progression of Head and Neck Cancer

**DOI:** 10.1155/2009/894064

**Published:** 2009-12-03

**Authors:** Zhuo (Georgia) Chen

**Affiliations:** Department of Hematology and Medical Oncology, School of Medicine, Winship Cancer Institute, Emory University, Atlanta, GA 30322, USA

## Abstract

Human head and neck cancer (HNC) is a highly heterogeneous disease. Understanding the biology of HNC progression is necessary for the development of novel approaches to its prevention, early detection, and treatment. A current evolutional progression model has limitations in explaining the heterogeneity observed in a single tumor nest. Accumulating evidence supports the existence of cancer stem cells (CSCs) as small subpopulations in solid tumors, including HNC. These CSCs can be selected by appropriate cell surface markers, which are cancer type specific and have been confirmed by unique *in vitro* and *in vivo* assays. Selected CSC populations maintain a self-renewal capability and show aggressive behaviors, such as chemoresistance and metastasis. In addition to introducing the CSC concept in solid tumors, this short review summarizes current publications in HNC CSC and the prospective development and application of the CSC concept to HNC in the clinic.

## 1. Introduction

Head and neck cancer is the sixth most common cancer and is responsible for almost 200,000 deaths around the world each year [1–3]. In the United States, head and neck squamous cell carcinoma (HNSCC) accounts for more deaths annually than cervical cancer, melanoma, or lymphoma. Although recent molecular studies have advanced our understanding of the disease and provided a rationale for the development of novel therapeutic strategies, HNSCC is still associated with severe mortality. Its 5-year survival rate has not been improved in more than 30 years [4]. In addition, the 5-year survival rate is even lower for HNSCC patients with a single homolateral lymph node metastasis (LNM) and is less than 25% for patients with bilateral LNM. Understanding the biology of HNSCC, progression will greatly assist in treatment decisions and in the development of new strategies for prevention and control of this disease.

Human neoplastic tumors, particularly HNSCC, are highly heterogeneous [5–7]. Currently, the progression of HNSCC is considered to result from evolution through stepwise alterations in multiple molecular and cellular pathways [8, 9]. However, this evolution concept has limitations in explaining the heterogeneity observed in a single tumor nest. It has been known for a long time that there are subpopulations of cells within solid tumors that contain different biological behaviors, such as metastatic potential [10, 11].

Accumulating evidence supports the subpopulation observation, particularly, the existence of so-called cancer stem cells (CSCs) [12–17]. Although CSCs in solid tumors including HNSCC have not been precisely identified, the CSC hypothesis opens a new era in understanding the initiation and progression of cancers. This short review will briefly introduce the CSC concept, summarize the current progress of CSC studies in HNSCC, and discuss the potential application of the CSC concept to the clinical management of HNSCC.

## 2. Cancer Stem Cell Concept

CSCs are defined as a small subset of cancer cells that constitute a pool of self-sustaining cells with the exclusive ability to maintain the tumor. Currently, there are two hypothetical explanations for the existence of CSCs. CSCs may arise from normal stem cells by mutation of genes that render the stem cells cancerous. Or, they may come from differentiated tumor cells that experience further genetic alterations and, therefore, become dedifferentiated and acquire CSC-like features.

The CSC concept is “an old idea reemerging at an important time” [12]. If the CSC hypothesis is true, many aggressive behaviors of cancer cells, such as chemoresistance and metastasis, may be better understood. Current CSC research is focusing on the identification of CSC in solid tumors, since stem cells in hematopoietic malignancies such as leukemia have been well characterized [12–16]. However, many difficulties are encountered when exploring the existence of CSCs in solid tumors, due to the inaccessibility of tumor cells and the lack of appropriate functional assays [17]. An important breakthrough in the study of solid tumor CSCs was the identification of breast cancer CSCs and their biomarkers by Clarke and his colleagues in 2003 [18]. Since then, CSCs have been reported in neoplasms of brain, prostate, lung, colon, pancreas, liver, melanoma, and skin [19–33]. Among them, the breast CSC model with well-defined biomarkers is more advanced than in other types of cancers [34–36]. Using this model, molecular signatures and signaling pathways have been further explored [34, 37].

There are three main characteristics that define CSCs: (1) differentiation, which provides the ability to give rise to a heterogeneous progeny, (2) self-renewal capability that maintains an intact stem cell pool for expansion, and (3) homeostatic control that ensures an appropriate regulation between differentiation and self renewal according to the environmental stimuli and genetic constraints of each organ tissue, which accounts for the tissue specificity of CSCs. Currently, xenograft assays for different organ sites have been established for testing CSCs. As suggested by the AACR Workshop on Cancer Stem Cells in 2006, the orthotopic xenograft assay is considered the golden standard for the identification of CSCs [12]. This type of assay allows reliable testing for all three characteristics of CSCs. In current studies, cancer cells from either tumor tissues or cell lines are initially sorted by specific cell surface markers. The selected cell population is then injected into experimental animals for tumorigenesis testing. If as few as 100–500 cells of the selected cell population are tumorigenic, the featured cell surface markers can serve as CSC-specific biomarkers. In a breast cancer study by Al-Hajj et al. [38], human breast cancer tissues or cells with or without expression of CD44 and CD24 were injected into the mammary fat pad of immune-deficient nonobese diabetic/severe combined immune-deficient (NOD/SCID) mice, which have greater immune deficiency than nude mice. Using this model, the breast CSC-specific biomarkers CD44^+^/CD24^−^ were determined. Similar xenograft assays in NOD/SCID mice were used to identify CSCs of brain, colon, and lung with a CD133^+^ profile [19, 21, 39–41]. Not only the NOD/SCID mouse models but also nude mice are choices for an orthotopic xenograft assay. Visvader and Lindeman have recently summarized mouse models and CSC markers used for isolation of CSC, including CD133, CD44, ALDH1A1, and epithelial cell adhesion molecule (EpCAM) [17]. As shown in Table 1, there is no universal CSC marker for all types of cancer. CSC markers may be tumor type specific, depending on the niche of each type of CSC. In addition to in vivo assays for CSC identification, many in vitro experiments have also provided evidence for the existence of CSCs. For example, studies by Collins et al. focused on a cell population in patients' tumor tissues featuring CD44^+^/integrin*α*2*β*1^high^/CD133^+^ [22]. These cells were examined by colony-formation and long-term serial culture assays and showed self renewal and regeneration of phenotypically mixed populations.

## 3. CSC-Related Cancer Progression Models

 Accumulating evidence suggests that CSCs contribute not only to tumor initiation, but also to aggressive tumor behaviors such as chemoresistance and metastasis.

### 3.1. CSC-Like Cells Constitute Part of a Chemoresistant Population

It has been noted that although chemotherapy kills the majority of cancer cells in tumor tissues, it may leave a population of cells behind. These cells overexpress the ATP-binding casstte (ABC) drug transporters which protect cancer cells from damage by cytotoxic agents. Coincidently, a side population (SP) of tumor cells which are defined by their inability to accumulate the fluorescent dye Hoechst 33342 due to overexpression of the ABC transporter ABCG2 has been confirmed to hold CSC features in several types of cancers including hematopoietic, prostate, and glioma CSCs [42–44]. ABCG2 and other ABC transporter proteins, therefore, have served as CSC markers [45] (Table 1). Chemoresistant activity has been identified in some CSC-like cell populations. For example, a study of a colorectal cancer cell line that is resistant to 5-fluorouracil (5FU) and oxaliplatin by Dallas et al. showed 5- to 22-fold enrichment of a double CSC marker CD133^+^/CD44^+^ population [46]. Another study by Hermann et al. showed that human pancreatic cells that survived prolonged treatment with gemcitabine had a 50-fold increase in a CD133^+^ population [32].

Considering CSCs a target population for the treatment of human cancer has opened new directions for research efforts in the field. The development of inhibitors against the ABC transporter ABCG2 has been explored in clinical studies [47]. On the other hand, targeting specifically activated signaling pathways in CSCs may provide an effective strategy to eliminate this cell population. Dallas et al. reported that chemoresistant colorectal cancer CSC-like cells showed increased expression of insulin-like growth factor-1 receptor (IGF-1R). This cell population responded to inhibition by an IGF-1R monoclonal antibody more effectively than its nonresistant counterpart [46]. Several signaling pathways, including the Wnt, TGF-*β*, and CXCR4 pathways, have been suggested to be activated in CSCs [17, 48, 49]. Therapeutically targeting these pathways deserves further investigation.

### 3.2. Migrating or Metastatic Cancer Stem Cells (mCSCs)

The existence of mCSCs was first hypothesized in 2005 by Brabletz et al., based on their observations in colorectal cancer [50, 51]. They proposed that there are two forms of CSCs in tumor progression—stationary CSC (sCSC) and mobile or migrating CSC (mCSC). They proposed that sCSCs are embedded in epithelial tissues or epithelial-based tumors and cannot disseminate. In contrast, mCSCs, which are derived from sCSC by acquiring a transient epithelial-mesenchymal transition (EMT), are located at the tumor-host interface and mediate tumor cell metastasis. In a colorectal cancer model, Brabletz et al. observed that not only the expression levels of EMT-related biomarkers but also their locations in the tumor nest were significantly associated with metastasis. They found that loss of E-cadherin (E-cad) usually resulted in nuclear localization of *β*-catenin, which is a typical feature of EMT, and nuclear *β*-catenin was accumulated in dedifferentiated tumor cells at the tumor-host interface. The authors then interpreted these observations in the context of the sCSC and mCSC hypotheses, suggesting that sCSC and mCSC are responsible for formation of the primary tumor and metastasis, respectively. Both sCSC and mCSC can lead to differentiation and tumor heterogeneity. Particularly, metastatic tumors generated from mCSC may experience a mesenchymal-epithelial transition (MET) in the metastatic organ site, which may explain why EMT can not be clearly observed pathologically in many metastatic lesions. In fact, the mCSC hypotheses can be used to explain the “heterogeneous morphology of the primary tumor and how metastases can recapitulate the heterogeneity in differentiation” and “tumor-cell dormancy and disease recurrence” [50]. Two recent publications support the mCSC hypotheses. Mani et al. reported that the stem-like cells identified in breast cancer were associated with EMT markers [49, 52]. A CD133^+^/CXCR4^+^ stem-like population isolated by Hermann et al. was suggested to be essential for metastasis of pancreatic cancer [32, 53].

### 3.3. Hierarchical and Stochastic Models of CSCs in Solid Tumors

**Figure 1:**
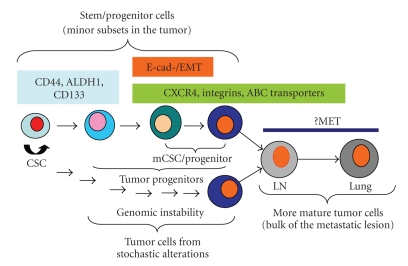
Hierarchical and stochastic models of CSC in progression of solid tumors.

Although the concept of developmental hierarchy of solid tumors has been discussed in several papers, the hypothetical hierarchical model of CSC/progenitors was clearly proposed in 2007 by Tang et al*. *based on their studies in prostate CSCs [43, 54]. This model described a hierarchical organization of phenotypically and functionally distinct cells at different stages of prostate tumor maturation. Their study demonstrated that a highly purified CD44^+^ population was still heterogeneous and enriched in tumorigenic and metastatic progenitors. That is, not only CSC but also progenitors can be tumorigenic in the NOD/SCID mouse model. These two types of tumor cells share the common marker CD44^+^, but they can be distinguished by other well-defined markers including ABCG2^+^ and *α*2*β*1^+^, which are specific for tumor progenitors. Recently, Odoux et al. identified chromosomal instability that usually supports a stochastic model in the mCSC population isolated from liver metastasis of colon cancer [55]. They, therefore, proposed a new model which suggested that both stochastic and hierarchical models can be used to explain the mCSC population (Figure 1).

## 4. CSC Studies in HNSCC

To date, only a few studies of HNSCC CSC have been reported [56]. Using both NOD/SCID mice and Rag2/cytokine receptor common *γ*-chain double knockout (Rag2*γ*DKO) mice, Prince et al., the same group that identified breast CSCs, reported that as few as 5 × 10^3^ CD44^+^ HNSCC cells could generate tumors in the mice and demonstrated tumor heterogeneity [57]. Examining samples from human HNSCC tissues revealed that the CD44^+^ population varied from 0.1% to 41.7%. This cell population also inclusively expressed BMI1, a nuclear protein that also plays a role in self renewal in other CSCs, while exclusively expressed the differentiation marker involucrin. Unlike breast CSCs, this group found that epithelial-specific antigen (ESA) expression was not enriched in the tumorigenic cells, suggesting that HNSCC has CSC biomarkers distinct from those in breast cancer. A CD44^+^ population was also reported by Okamoto et al. to characterize HNSCC CSC-like cells [58]. It was found that CD44^+^** cells possessed not only a capacity for forming tumor spheres, proliferation, migration, and invasion in vitro, but also a resistance to chemotherapeutic agents. Supporting this observation, four relevant chemoresistant genes, *ABCB1*, *ABCG2*, *CYP2C8*, and *TERT*, were upregulated in the CD44^+^ population. Recently, an SP was identified by Zhang et al., and proved to enhance the capability of tumor formation in nude mice as compared with non-SP [59]. In another study, oral cancer stem-like cells were enriched through sphere formation and found to express Oct-4, Nanog, CD133, and ABCG2 [60]. Nanog/Oct-4/CD133 triple-positive status predicted a poor prognosis for patients with oral cancer. CD133 is also reported as an HNSCC stem-like cell marker by studies using a head and neck cancer cell line [61]. These data can be supported by many observations showing that a small population of HNSCC tumor cells exists and demonstrates strong self-renewal and proliferation capabilities, even in the early stage of tumor development [62–64]. In tumor cells of epithelial origin, this subpopulation shows a dedifferentiation phenotype and plasticity, which facilitates metastasis of HNSCC. In fact, this tumor subpopulation is also responsible for more aggressive phenotypes, such as resistance to cancer therapeutic drugs and metastasis [50, 51].

Whether putative CSCs play a role in metastasis of HNSCC or not the existence of mCSC has not been reported. But our previous study provides indirect evidence supporting the existence of such a population. We found that a highly metastatic subpopulation selected from a xenograft mouse model expressed high levels of CSC markers, including CXCR4 and integrin *β*1, and altered levels of EMT markers such as E-cadherin and vimentin [65–67]. CXCR4 has been investigated as a putative CSC marker and is also an ideal target for the treatment of metastatic HNSCC. Integrin *β*1 is mainly expressed in the basal layer of the normal epithelium as an epithelial stem cell marker [64, 68]. In abnormal epithelium (hyperplasia and dysplasia), integrin *β*1 is found to be expressed in the upper layers of the epithelial tissues. It is also expressed in a variety of tumor tissues. Integrin *β*1 overexpression has been suggested to expand the CSC compartment by inhibiting differentiation and apoptosis, therefore contributing to tumor progression and metastasis [68]. A recent study by Kirkland and Ying showed that *α*2*β*1 integrin regulated lineage commitment in multipotent human colorectal cancer cells [69]. Whether the metastatic populations contain CSC-like features or not is currently under investigation.

## 5. Implications of CSC in the Development of Biomarkers and Therapy for HNSCC

From a clinical perspective, if the CSC or CSC-like population represents the more aggressive HNSCC population, the early detection and targeted treatment of these cells become an urgent need in order to better manage this disease. CSC-specific markers provide unique tools for identifying these putative aggressive cell populations. An immunohistochemistry study of primary HNSCC reported by Prince and Ailles showed that CD44 staining was associated with more basal-appearing cells [56]. CD44^+^ cells were costained with markers for the basal normal squamous epithelium, CK5/14, while CD44^−^ cells were associated with the differentiation marker involucrin, supporting the organization of HNSCC by developmental hierarchy, as predicted by the CSC theory of carcinogenesis. However, some studies of CD44 as a CSC marker in human HNSCC tissues contradict these in vitro and in vivo studies. A recent study by Mack and Gires reported CD44s and CD44v6 expressions in head and neck epithelial tissues [70]. They found a similarly high level of CD44s and CD44v6 expression in normal, benign, and malignant epithelia of the head and neck. A similar observation was also obtained in our laboratory (data not shown). Therefore, the value of CD44s as a marker for a small CSC population in HNSCC needs to be reconsidered. We believe that there is a necessity to precisely define more HNSCC CSC markers with an aim of further improving our ability to isolate HNSCC CSCs.

**Figure 2:**
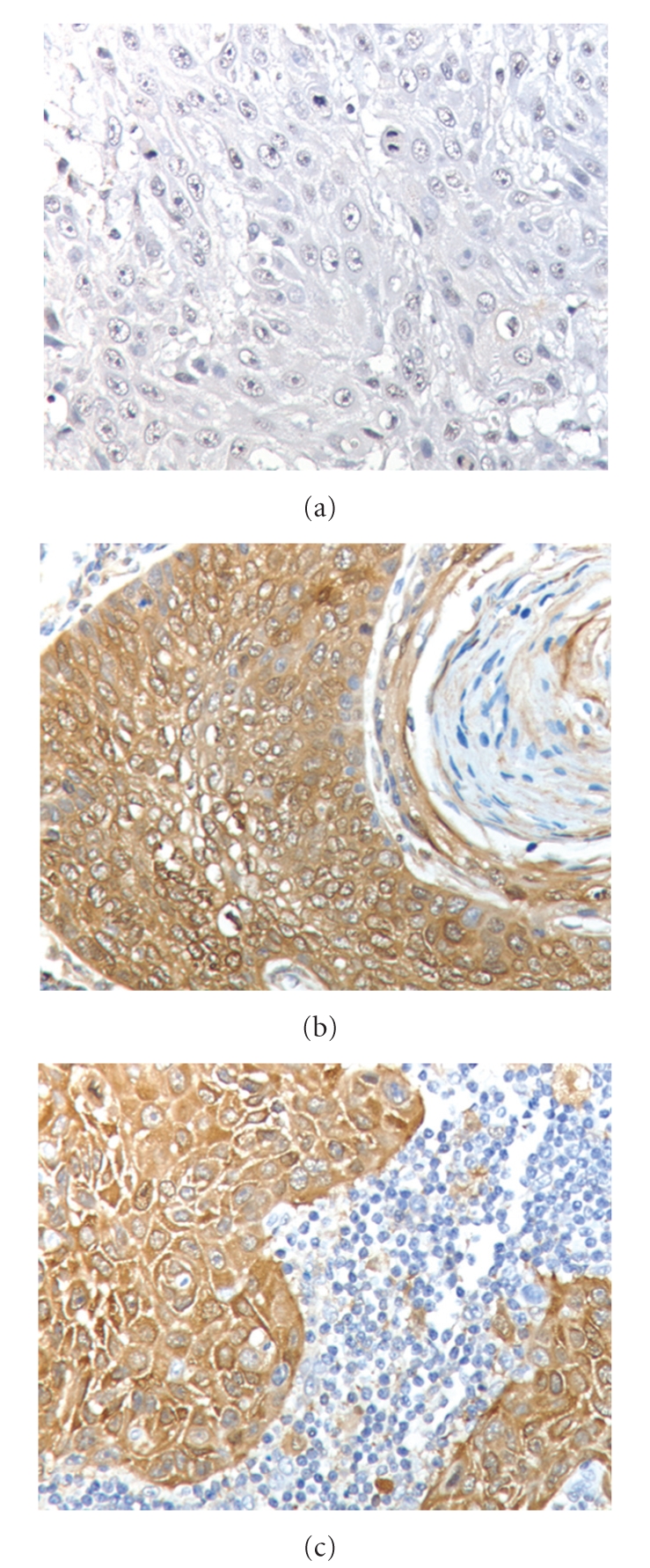
ALDH1 expression in HNSCC tissues: (a) nonmetastatic primary tumor with negative ALDH1 expression, (b) metastatic primary tumor with positive ALDH1 expression, and (c) corresponding lymph node metastases. (Magnification: 400x).

Another possible CSC marker expressed in HNSCC is ALDH1. ALDH1 has been considered a marker of normal and malignant human mammary stem cells and a predictor of poor clinical outcome [71]. Expression of ALDH1 in HNSCC and dysplastic mucosa tissue samples was examined by Visus et al. [72]. They found that 12 of 17 HNSCC and 30 of 40 dysplastic mucosa tissues expressed this protein. However, this study did not correlate ALDH1 expression status with aggressiveness or prognostic features of the disease, such as metastasis, chemoresistance, or survival. Our recent study of HNSCC tissues demonstrated a statistically significant increase in ALDH1 expression in tumors with LNM compared to tumors without LNM (*P* < .0003, Figure 2). Although ALDH1 has not been reported as a marker for HNSCC CSC, our study suggests that ALDH1 may be a potential marker for tumor progression and metastasis in HNSCC.

In addition to their predictive and prognostic value, the identification of CSCs in HNSCC will also provide target populations that require more aggressive treatment than can be achieved with conventional therapies, such as a combination treatment with chemotherapy and an agent targeting CSC-specific signaling pathways. As discussed in Section 3.1., a combination of chemotherapy with inhibitors of the ABC transporters overexpressed by CSCs may have potential clinical application. Furthermore, recent progress in nanotherapeutics has shown the ability of nanoparticles to bypass ABC transporters when delivering anticancer drugs to tumor cells, providing a new strategy to overcome chemoresistance of CSCs [73].

## 6. Conclusions

Recent progress in the study of CSCs in solid tumors has provided researchers and clinicians in head and neck cancer new concepts to better understand the heterogeneity of this disease with. Once CSC or CSC-like populations are defined with appropriate biomarkers, these biomarkers can be used for accurately detecting tumor-initiating cells or metastatic cells in primary tumor biopsies, which will aid clinicians in their treatment decisions and in the accurate prognosis of HNSCC.

Currently, there are no consistently well-defined biomarkers or matured technologies to identify CSC or CSC-like populations in HNSCC. Efforts are being made to improve this situation by developing in vitro models and appropriate HNSCC CSC culture systems and refining techniques for the selection of well-defined cell populations from clinical samples. Furthermore, major signaling pathways in CSC or CSC-like populations of HNSCC are under investigation. The major cellular signaling mediators should be ideal targets for the development of new therapeutic agents to specifically eradicate high-risk HNSCC cells, which may also hold drug-resistant phenotypes. These studies are part of a growing interest toward personalized treatment for HNSCC.

## Figures and Tables

**Table 1:** Putative CSC makers in solid tumors.

CSC markers	Tumor types	% CSC markers	Minimal cell no.	Refs
		in tumor cells	for tumor formation	
CD44^+^/CD24^−/low^	Breast	11–35	200	[18]
CD44^+^	Head and neck	0.1–42	5000	[57]
	Prostate	0.3–38	100	[26]
CD44^+^/EpCA*M* ^hi^	Colon	0.03–38	200	[31]
CD44^+^/CD24^−^/ESA^+^	Pancreas	0.2–0.8	100	[27]
ALDH1^+^	Breast	3–10	500	[71]
CD133^+^	Brain	6–29	100	[21]
	Brain	2-3	500	[39]
	Colon	1.8–25	200	[40]
	Colon	0.7–6	3000	[27]
	Head and neck	0.8–4.2	1000	[60]
	Pancreas	1–3	500	[32]
	Lung	0.32–22	10^4^	[19]
Side population	Prostate	0.05–0.2	100	[33]
ABCG5^+^	Melanoma	1.6–20	10^6^	[30]
